# Roles of protein tyrosine phosphatases in hepatocellular carcinoma progression (Review)

**DOI:** 10.3892/or.2023.8485

**Published:** 2023-01-19

**Authors:** Yi-Li Chen, Ching-Chuan Hsieh, Pei-Ming Chu, Jing-Yi Chen, Yu-Chun Huang, Cheng-Yi Chen

**Affiliations:** 1 Department of Cell Biology and Anatomy, College of Medicine, National Cheng Kung University, Tainan 70101, Taiwan, R.O.C.; 2 Division of General Surgery, Chang Gung Memorial Hospital, Chiayi 613, Taiwan, R.O.C.; 3 Department of Anatomy, School of Medicine, Chung Shan Medical University, Taichung 40201, Taiwan, R.O.C.; 4 Department of Medical Education, Chung Shan Medical University Hospital, Taichung 40201, Taiwan, R.O.C.; 5 Department of Medical Laboratory Science, College of Medicine, I-Shou University, Kaohsiung 82445, Taiwan, R.O.C.; 6 School of Medicine for International Students, College of Medicine, I-Shou University, Kaohsiung 82445, Taiwan, R.O.C.; 7 Aging and Diseases Prevention Research Center, Fooyin University, Kaohsiung 83102, Taiwan, R.O.C.

**Keywords:** protein tyrosine phosphatases, hepatocellular carcinoma, drug resistance, inflammation

## Abstract

Hepatocellular carcinoma (HCC) represents almost 80% of all liver cancers, is the sixth most common cancer and is the second-highest cause of cancer-related deaths worldwide. Protein tyrosine phosphatases (PTPs), which are encoded by the largest family of phosphatase genes, play critical roles in cellular responses and are implicated in various signaling pathways. Moreover, PTPs are dysregulated and involved in various cellular processes in numerous cancers, including HCC. Kinases and phosphatases are coordinators that modulate cell activities and regulate signaling responses. There are multiple interacting signaling networks, and coordination of these signaling networks in response to a stimulus determines the physiological outcome. Numerous issues, such as drug resistance and inflammatory reactions in the tumor microenvironment, are implicated in cancer progression, and the role of PTPs in these processes has not been well elucidated. Therefore, the present review focused on discussing the relationship of PTPs with inflammatory cytokines and chemotherapy/targeted drug resistance, providing detailed information on how PTPs can modulate inflammatory reactions and drug resistance to influence progression in HCC.

## 1. Introduction

Hepatocellular carcinoma (HCC) is the most common type of liver cancer, accounting for ~90% of all cases (1). HCC is one of the most common malignancies and the third leading cause of cancer-related death (2). Over 4 million estimated new cases of HCC have been reported, and HCC has been reported to cause over 3 million estimated deaths in USA (3). Multiple factors, including hepatitis B and C, obesity, excess ingestion of alcohol and smoking, lead to HCC progression. Apart from external factors, gene deficiency or mutation is also recognized as a potential cause of liver cancer progression (4–8). General treatments for HCC are surgery, liver transplantation, and drug therapy. However, the overall efficacy of HCC treatment remains unsatisfactory due to the high recurrence and progression rates (9). Therefore, ongoing studies in HCC are necessary. Complex molecular signaling pathways associated with cell proliferation, metastasis, inflammatory reactions and drug resistance are involved in HCC. The multiple types of molecules involved in these pathways interact with each other and provide various signal transduction pathways that are highly activated in cancers (10–13).

HCC is the most common primary hepatic malignant tumor and has been the 2nd most common cause of cancer-related years of life lost worldwide. The prevalence in Taiwan ranks 4th among all malignancies (~29/100,000) and it is the 2nd cause of mortality (14). Most individuals diagnosed with HCC often have chronic liver disease, particularly chronic hepatitis B and C infection, and have not always symptoms in the early stage. ~70% of patients cannot receive curative therapy when they are diagnosed. The average survival time of patients with HCC in Barcelona Clinic Liver Cancer (BCLC) stage C is ~11 months, and that of patients in stage D is ~3 months.

In 2007, sorafenib was approved for HCC treatment. The therapy of HCC entered a new era compared with traditional chemotherapy, and sorafenib became a standard of care in the BCLC stage C group. In the pivotal sorafenib HCC assessment randomized protocol (SHARP) trial, sorafenib in patients with BCLC stage C HCC showed an increased 2.8-month overall survival (OS) (10.7 months) compared with placebo (7.9 months) (15). Before sorafenib, no significant trial had shown a favorable response to treat patients with HCC by traditional chemotherapy. Sorafenib is a multikinase inhibitor (MKI) that suppresses tumor cell proliferation and angiogenesis through different pathways (16). However, in the last 10 years, other TKIs have failed to improve the efficacy of sorafenib, including erlotinib, brivanib, sunitinib and everolimus. Until 2017, regorafenib (REG) showed a significant treatment response in patients with sorafenib-refractory disease compared with placebo. In recent years, several TKIs have shown treatment effects as 2nd-line (REG, cabozantinib, ramuciramab) and 1st-line (lenvatinib) agents, and the development of immune checkpoint inhibitors (ICIs) has revealed another treatment option. Systemic therapy for the BCLC stage C group or BCLC stage B group refractory to transarterial embolization had more medication choices in those years (17).

HCC patients with moderately compromised liver function (Child-Pugh B functional status) have limited curative options due to the risk of liver failure (18). This is particularly evident in HCC cases not suitable for surgery or locoregional treatments. For these patients, the availability of sorafenib differs, which is differentiated by the different international guidelines and local regulatory policies (19). Previously, the potential mechanisms of metronomic capecitabine (MC) have been mentioned, including blockage of tumor angiogenesis, reduced therapeutic resistance, and activation of immune responses (20–22). De Lorenzo *et al* (23) reported that the median OS of 35 MC-treated patients was 7.5 months [95% CI: 3.733-11.267] and 5.1 months [95% CI: 4.098-6.102] in the 70 BSC group (P=0.013) (23). Furthermore, 12 patients (34.3%) treated with MC experienced several adverse events, including fatigue (17.1%), hand-foot syndrome (8.5%), neutropenia (5.7%), and thrombocytopenia (8.5%). However, MC appears to be a safe choice for Child-Pugh B-HCC patients. Its potential antitumor activity warrants prospective evaluations (23). Similarly, MC also displayed a therapeutic alternative for CP-B patients who were resistant to tyrosine kinase inhibitors (TKIs).

Furthermore, radiation, chemotherapy, TKIs and ICIs as adjuvant strategies have not been demonstrated to improve the clinical status of progression-free survival (PFS) or OS in resected early-stage disease (24). Patients with early recurrence risk are usually associated with negative prognostic factors, whereas those at risk of late recurrence are always associated with advanced liver disease, such as tumor formation (25). In fact, some studies have shown that adjuvant systemic therapy can reduce local and distant recurrence rates; however, more recent studies have not demonstrated this effect (26).

Previously, Ohata *et al* (8) evaluated whether adjuvant systemic treatment could improve survival in early-stage HCC, and the STORM trial evaluated the effect of adjuvant sorafenib in HCC. In this phase 3, double-blind study, 1,114 patients were randomly assigned to placebo or sorafenib-stimulated groups, who were treated with 400 mg twice a day for up to 4 years. However, relapse-free survival was not significantly different between the two groups (27). Based on the evidence, it was considered that the efficacy of anticancer agents cannot translate from the advanced to the adjuvant setting. Unfortunately, effective regimens thus far have not been found. At the same time, because TKIs need an optional ‘target’ to perform their activity, it was suggested that these drugs in the adjuvant setting are probably far from being rational (10). Therefore, immunotherapy is considered to be a promising approach, and patients should be encouraged to enroll in clinical trials evaluating immune-based combinations or ICI monotherapy as adjuvant treatment to reduce the risk of recurrence and improve clinical outcomes.

REG, an orally bioavailable MKI, blocks the activity of several protein kinases, including KIT, BRAF, RAF-1, RET, VEGFR-1, VEGFR-2, VEGFR-3, PDGFR and FGFR, which are associated with tumor microenvironment signaling, tumor angiogenesis, and cell proliferation (28,29). Currently, REG is approved as a single agent for the treatment of HCC at a dose of 160 mg orally once daily on days 1–21 of each 28-day cycle (30). There are currently ongoing trials aimed at evaluating the efficacy of REG as monotherapy or in combination with other anticancer agents, and the number of patients receiving REG is expected to increase in the future (31,32). Therefore, it was suggested that REG dose personalization may improve quality of life, decrease treatment adverse events, and enhance patient outcomes.

ICIs are able to influence immune checkpoint-related molecules, such as programmed cell death-1 (PD-1), cytotoxic T-lymphocyte-associated antigen 4 (CTLA-4), and lymphocyte-activation gene 3 (LAG-3) (33,34). However, ICI monotherapy has demonstrated disappointing effects thus far in patients with HCC (35,36). Recently, combined immunotherapy with phase III IMbrave150 and the PD-L1 inhibitor atezolizumab plus anti-angiogenesis bevacizumab showed more convincing effects than monotherapy in advanced HCC patients (37,38). Moreover, patients receiving another combined immunotherapy, including atezolizumab and bevacizumab, had impressive benefits in PFS, OS, objective response rate, and complete response rate, and the median OS could reach 19.2 months (39). However, to date, few molecules have been assessed as predictors, such as programmed death ligand-1 (PD-L1), tumor mutational burden, and gut microbiota, to evaluate whether these therapeutic regimens benefit patients with HCC (40). Hence, it was suggested by the authors that a useful marker is needed to evaluate whether monotherapy or combined immunotherapy benefits patients with HCC.

The review entitled ‘The Roles of Protein Tyrosine Phosphatases (PTP) in HCC’ published in 2018, reported both oncogenic and tumor suppressive function of PTPs in HCC. Huang *et al* (41) mainly reviewed the involvement of PTP and associated signaling pathways in HCC. However, the present review mainly focused on discussing the relationship of PTPs with inflammatory cytokines and chemotherapy/targeted drug resistance, providing detailed information on how PTPs can modulate inflammatory reactions and drug resistance to influence progression in HCC. The roles of inflammatory cytokines and drug resistance modulated by PTPs have never been displayed previously. Therefore, it was suggested that the PTP-regulated inflammatory cytokines and drug resistance are critical in HCC, and PTPs may be therapeutic targets in the future. In the present review, numerous kinds of PTPs associated with cancer progression were discussed, including those associated with inflammatory reactions and drug resistance in HCC.

## 2. The PTP family

Class I PTPs include ~100 proteins, which can be separated into two groups based on their interaction residues: classical PTPs and dual-specificity phosphatases (DSPs). The classical PTPs include ~38 proteins; these proteins are dispersed in the cytoplasm (non-transmembrane PTPs) and cellular membrane (receptor-like PTPs), and the classical PTPs are involved in a broad range of pathways connecting intracellular and extracellular components (42).

Tyrosine phosphate groups are the general site of dephosphorylation of classical PTPs. By contrast, DSPs have three phosphate-related areas, including tyrosine, serine and threonine residues. In addition to DSPs having smaller catalytic domains than classical PTPs, there are other major differences between classical PTPs and DSPs. Most DSPs are localized in the cytoplasm, and due to the diverse phosphate residues involved in the dephosphorylation process, DSPs regulate various cellular biogenesis processes, including the cell cycle, metabolism and neuron transduction (43). Currently, DSPs are categorized into several subclasses, such as mitogen-activated protein kinase (MAPK) phosphatases (MPKs, 11 members), protein phosphates slingshot homologs (3 members), phosphatases of regenerating liver (PRLs, 3 members), phosphate and tension homologs (PTEN, 5 members), atypical DSPs (19 members), myotubularins (16 members), and CDC14s (4 members) (Table I) (43,44). To date, certain of these DSP subclasses have been revealed to potentially regulate pathways in multiple diseases, particularly cancer. The MAPK/extracellular signal-regulated kinase (ERK) pathway is one of the most important pathways, and its members have been identified to be overexpressed or mutated in over 90% of cancers. The target phosphatases of this pathway are MPKs. Moreover, cancer cells are able to regulate CDK-induced cell cycle progression through regulation of CDC14s; a previous study has revealed upregulation of certain PRLs and downregulation in certain PTEN members and myotubularins (45). Most class I PTPs induce profound effects in cancers, while the characteristics of other genes and proteins remain unclear (44). Low-molecular-weight phosphatase, also called ACP1, is the only member of the class II PTPs, and it has been demonstrated to be associated with platelet-derived growth factor signaling (46).

## 3. PTP mechanisms

PTPs are multifunctional enzymes mainly localized in the cytoplasm and cellular membrane. These enzymes play an essential role in protein post-translational modification, which affects protein activity and stability (47,48). A common mechanism of PTPs is to recognize the phosphorylation residues on tyrosine, serine, and threonine residues of target proteins to induce the removal of phosphate groups, leading to the dephosphorylation and inactivation of kinases or receptors (49). Two PTP domains participate in this process, the PTP-loop and WPD-loop. In the initiation of the phosphorylation catalytic process, the cysteine catalytic site on the PTP-loop competes for the binding of the phosphate group and the target protein, which results in breaking of the phosphate-oxygen bond and dephosphorylation of the target residue. Concurrently, the WPD loop stabilizes the remaining structure. In the further steps of the catalytic process, the phosphate group is released via cooperation of the P-loop and WPD-loop (50).

Protein tyrosine phosphorylation is a pivotal process for signal transduction in eukaryotic cells and is a reversible regulatory mechanism that is coordinately controlled by protein tyrosine kinases (PTKs) and PTPs (51). Imbalance of the PTK-PTP axis often results in aberrant protein tyrosine phosphorylation in cancers and promotes tumorigenesis, as is observed in HCC (52). PTPs usually play tumor-suppressor roles, whereas PTKs are mainly associated with oncogenic and tumorigenic activities (53). Regulation of RTKs and PTPs, which occurs by reversible alteration of the phosphorylation state of specific tyrosine kinases, leads to various cellular events, such as alterations in the signaling pathway activity and cellular phenotypes (54).

## 4. Association between PTPs and cytokines in HCC

**Figure 1.**
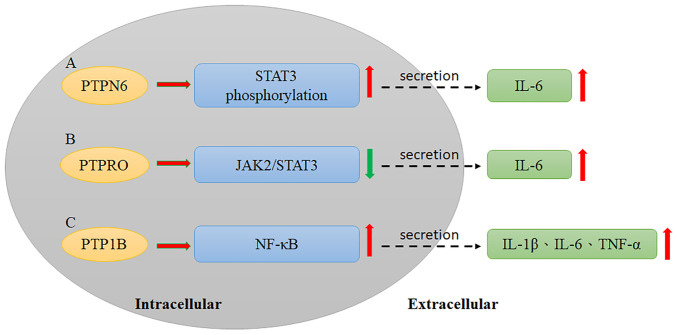
PTPs modulate cytokine secretions via various signaling pathways. (A) PTPN6 and (B) PTPRO can induce IL-6 secretion via upregulated STAT3 phosphorylation and downregulated JAK2/STAT3 pathways, respectively. (C) PTP1B can activate NF-κB signaling to induce IL-1β/IL-6 and TNF-α secretions. PTPs, protein tyrosine phosphatases; PTPN6, protein tyrosine phosphatase, non-receptor type 6; PTPRO, protein tyrosine phosphatase receptor type O.

Cytokines have been identified as potential factors in cancer progression and are components of the tumor microenvironment. Cytokines include a broad range of extracellular molecules, such as chemokines, interleukins, interferons and tumor necrosis factors (TNFs). They are generally produced in the extracellular environment by immune cells, endothelial cells and stromal cells and regulate downstream cellular signaling by binding to specific receptors (55). Due to the enormous variety of cytokines and their varied characteristics, the final cellular effects of cytokines are usually substantial and long lasting. Cytokines can also increase cancer cell malignancy by modifying cellular proliferation, migration and invasion (55). Several studies have demonstrated that some cytokines and PTPs interact in HCC progression. PTPN6 interacts with gankyrin, an oncoprotein, leading to the secretion of IL-6, which induces signal transducer and activator of transcription 3 (STAT3) phosphorylation and promotes HCC development (56); Sakurai *et al* (56) reported that gankyrin can interact with PTPN6 to induce STAT3 activation and IL-6 secretion. Moreover, gankyrin can also increase VEGF expression, which leads to HCC progression (Fig. 1A, Table II) (56). however, more studies have identified PTPs as potential candidates for reversing cytokine-induced HCC progression. PTP receptor type delta (PTPRD) is a tumor suppressor that is negatively correlated with PD-L1, an essential molecule in cancer immune escape. Overexpression of PTPRD is able to reduce PD-L1 through the STAT3 pathway activation (Table II) (57). In addition, the effects of combination therapies also involve PTP-cytokine relationships. Induction of SHP-1 through crocin decreases the IL-6-stimulated STAT3 pathway, resulting in HCC cell apoptosis (58). Quercetin enhances IFN-α-induced phosphorylation of STAT1 by downregulating SHP2, leading to an anti-proliferative effect in HCC (59).

In addition to classical PTPs, DSPs are a subgroup of tyrosine phosphatases in the PTP family, and these enzymes function in the removal of a wide range of phosphate groups from not only tyrosine residues but also serine/threonine residues (44). Similar to classical PTPs, DSPs are involved in broad signaling transduction in the regulation of the development of both normal and cancer cells, and these proteins also exhibit potential relationships with cytokines (60). Sorafenib, the most common targeted therapy in HCC treatment, induces DSP1 expression and reduces TGF-β expression in macrophages, potentially promoting HCC progression (61). Moreover, knockdown of CDC25A decreases the expression of IL-6 and IL-1β, leading to significant cell cycle arrest in the G1 phase in HCC (62).

Previously, PTP receptor type O (PTPRO) and its truncated form (PTPROt) were defined as negative regulators of JAK2/STAT3 signaling (63). Hou *et al* (63) reported that PTPRO downregulates STAT3 activation via the JAK2 and PI3K signaling pathways. Therefore, the effect of PTPRO on HCC development may result from STAT3 activation. Numerous studies have reported that the poor therapeutic effect of PD-1 adjuvant treatment is highly associated with an increased level of IL-6 in serum. Anti-PD-1/PD-L1 antibody combined with anti-IL-6 antibody treatment has demonstrated significant curative effects in animal models (64). Therefore, IL-6 may have a crucial impact on the effect of PD-1/PD-L1 adjuvant therapy. JAK2 has been demonstrated to interact with PTPRO upon IL-6 stimulation by a coimmunoprecipitation assay, indicating that PTPRO may modulate JAK2 (Fig. 1B, Table II). PD-L1 expression in monocytes and macrophages has been demonstrated to be suppressed by PTPRO through downregulation of the JAK2/STAT1 and JAK2/STAT3/c-MYC cascades (65).

PD-L1 expression was significantly increased in PTPRO-expressing macrophages in an IFN-γ-dependent manner after IL-6 treatment for 72 h. This result could indicate that PTPRO expression is essential for IL-6-induced Pd-L1/PD-L1 expression in both *Ptpro* knockout and PTPRO knockdown macrophages. Moreover, signaling pathway analysis indicated that the action of PTPRO is dysregulated by IL-6 via increased activation of the STAT3/c-MYC/PD-L1 axis in monocytes and macrophages. Treatment of U937- and THP-1-derived macrophages with c-MYC shRNA reversed the IL-6-induced decrease in PTPRO expression (65). IL-6 is secreted by both T cells and macrophages and is a classic proinflammatory cytokine. IL-6 is a key cytokine linking inflammation to tumorigenesis in numerous cancers, including HCC (66,67). Moreover, Naugler *et al* (68) also reported that IL-6 is an essential cytokine linking inflammation and tumorigenesis. IL-6 has been demonstrated to play an oncogenic role in obesity-related HCC (69).

The nonreceptor PTP SHP2, encoded by PTP, nonreceptor type 11 (PTPN11), is a critical member of the RAS/ERK pathway and most receptor tyrosine kinase, cytokine receptor, and integrin signaling pathways (70). Several lines of evidence have indicated that PTPN11 is involved in HCC progression (71). In addition, several studies have shown that PTPN11 can also play an unexpected tumor suppressor role in HCC (72,73), implying that PTPN11 possesses dual roles in tumorigenesis.

PTPRD, a member of the PTP family, has been reported to act as a tumor suppressor gene and plays a crucial role in controlling numerous cellular processes, including cell proliferation, apoptosis, survival and motility (74). PTPRD is often inactivated via deletion or epigenetic mechanisms in several cancers (75). STAT3, a major transcription factor, is involved in numerous cellular processes, such as cell growth, proliferation, migration, differentiation and death (76). Previously, STAT3 has been demonstrated to positively regulate PD-L1 expression to promote immune escape in cancer (77). Furthermore, Meng *et al* (57) reported that PTPRD expression was significantly lower in tumor tissues than in normal tissues; however, PD-L1 was significantly overexpressed in cancer tissues compared with normal tissues.

Additional studies have also shown that silencing PTP1B decreases the inflammatory response and levels of associated cytokines, including IL-1β, IL-6 and TNF-α, while overexpression of PTP1B induces inflammation in RAW264.7 cells. Moreover, lipopolysaccharide can activate the NF-κB pathway in RAW264.7 cells, and NF-κB signaling is also affected by dysregulated PTP1B expression (Fig. 1C, Table II) (78).

The nonreceptor PTP Src homology region 2 (SH2) domain-containing phosphatases (SHPs), including SHP-1 (also known as PTPN6) and SHP-2 (also known as PTPN11), are critical modulators of numerous fundamental cellular processes, such as cell proliferation, differentiation and inflammation (79). SHP-2 is a ubiquitously expressed modulator of inflammatory reactions and is implicated in HCC carcinogenesis and progression (80). In addition, SHP-1 is extensively expressed in hematopoietic and epithelial cells and is widely defined as a negative regulator of inflammation (81). Several studies have reported that MKIs, such as sorafenib (82) and dovitinib (83), exert their antitumor effects by enhancing SHP-1 phosphatase activity. TGF-β1-induced STAT3 (Tyr705) phosphorylation and epithelial-to-mesenchymal transition can be abolished with SHP-1 overexpression, which blocks cell migration and invasion of HCC (84). Moreover, SHP-1 has been demonstrated to be overexpressed in non-cancer tissues compared with surrounding cancer tissues, and reduced SHP-1 expression is highly associated with poor prognosis of patients with HCC (85). Collectively, SHP-1 can be defined as a tumor suppressor that prevents the initiation and progression of HCC in animal models (85). SHP-1 can also inhibit the activation of various signaling pathways, such as the STAT3, NF-kB, and AKT pathways, to suppress hepatocarcinogenesis and the malignant phenotype of HCC (85). Several drugs, including sorafenib, dovitinib, and SC-2001, induce cell apoptosis and inhibit the growth of HCC cells by enhancing the activity of SHP-1 tyrosine phosphatase (82,86). SHP-1 and SHP-2, cytoplasmic PTPs, share similar sequences, containing two Src homology 2 (SH2) NH2-terminal domains and a C-terminal protein-tyrosine phosphatase domain (87). SHP-2 reduces STAT3 phosphorylation via the JAK/STAT pathway to suppress HCC initiation. By contrast, SHP-2 coordinately activates the Ras/Raf/Erk and PI3K/Akt/mTOR cascades to promote the progression of HCC (80). Liver inflammation, a primary oncogenic factor, is highly associated with HCC (88,89). The inflammatory cytokines induced by liver injury activate inflammatory signaling pathways, including the JAK/STAT and NF-kB signaling pathways, which in turn induce the expression of IL6, TGF-β, and TNF-α (90,91).

## 5. The relationship between PTPs and drug resistance

**Figure 2.**
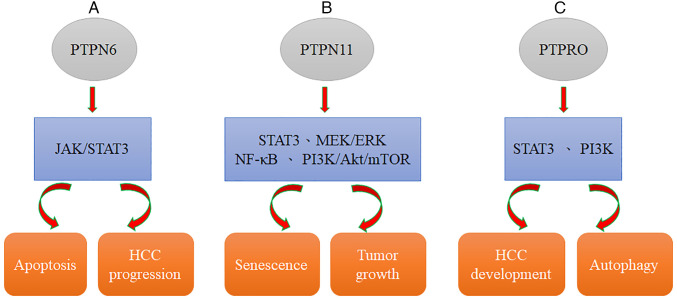
PTPs regulate drug resistance through numerous signaling pathways. (A) PTPN6 induces cell apoptosis and HCC progression through JAK/STAT3 signaling. (B) PTPN11 influences cell senescence and tumor cell growth via the STAT3/MEK/ERK/NF-κB and PI3K/Akt/mTOR pathways. (C) PTPRO regulates HCC development and cell autophagy via the STAT3 and PI3K pathways. PTP, PTPs, protein tyrosine phosphatases; PTPN6, protein tyrosine phosphatase, non-receptor type 6; PTPN11, protein tyrosine phosphatase, non-receptor type 11; PTPRO, protein tyrosine phosphatase receptor type O; HCC, hepatocellular carcinoma.

SHP2 is related to the stress sensor DNA damage 45G (GADD45G), which is involved in multiple biological processes and downregulated in various cancers. GADD45G has been demonstrated to induce senescence in HCC and reduce tumor growth *in vivo.* Moreover, GADD45G-induced senescence can be efficiently counteracted with Shp2 silencing. GADD45G expression is negatively correlated with the phosphorylation status of STAT3 in tumor cells of clinical HCC specimens (Table III) (92); this result is related to the relationship of SHP2 with STAT3 (93,94). Dovitinib downregulation of p-STAT3 and induction of apoptosis can be abolished by using an SHP-1 inhibitor or silencing SHP-1 with RNA interference, suggesting that SHP-1, a PTP, modulates the effects of dovitinib. In addition, dovitinib reduced STAT3 activation to induce cell apoptosis in two sorafenib-resistant cell lines, and sorafenib-resistant cells showed significant activation of STAT3, indicating that STAT3 may be a useful target to overcome drug resistance in HCC (54). JAK/STAT3 signaling is inactivated by several PTPs, including the SH2 domain-containing cytosolic phosphatases SHP-1 and SHP-2 (95,96). Furthermore, SHP-1 has been demonstrated to be involved in the dovitinib-mediated effect on kinase inhibition, phosphorylated (p)-STAT3 and apoptosis in HCC (54). Additionally, dovitinib suppressed tumor growth in both Huh-7 and PLC5 ×enograft tumors *in vivo*, suggesting the potential utility of dovitinib in the clinical practice. Therefore, an understanding of the mechanism of SHP-1-mediated STAT3 inhibition provides a potential target for future HCC molecular therapy (Fig. 2A, Table III) (54).

SHP2, encoded by PTPN11, was found to not only be overexpressed in HCC (97) but also serve as a potential predictive biomarker for sorafenib response and patient survival (97). Moreover, SHP2 has been defined as a downstream effector of numerous RTKs, and SHP2 blockade may be a possible mechanism causing RTK activation, resulting in the development of acquired resistance to sorafenib in HCC (98). Collectively, sorafenib-induced reactivation of the RTK-mediated AKT and MEK/ERK pathways can be significantly induced by SHP099 (98). Targeting SHP2 with SHP099 combined with sorafenib treatment may be a novel and safe therapeutic strategy against HCC (99). Previously, Kang *et al* (100) reported that the RNA level of SHP2 is upregulated through the NF-κB signaling pathway in HBX-transfected HCC cells. Mechanistically, SHP2 expression is induced by direct binding of NF-κB to its promoter. Since NF-κB signaling has been implicated in HCC progression (100) and sorafenib resistance (101), its activation may be a potent mechanism leading to SHP2 upregulation in both parental and sorafenib-resistant HCC cells. Previously, SHP2 was identified as an oncogenic tyrosine phosphatase that contains two Src-homology 2 domains (102). SHP2 has been reported to be a critical component of multiple RTK signaling pathways activated in response to numerous growth factors, including FGFR, EGFR, PDGFR, and VEGFR, and this activation leads to induction of the PI3K/AKT/mTOR pathway and ERK signaling based on genetic and biochemical evidence (103,104). SHP2 is required for RTK-evoked RAS activation, which results in the activation of the MEK/ERK and AKT pathways (Fig. 2B, Table III) (105).

SHP-1 is a PTP that is largely expressed in hematopoietic cells. To date, several studies have addressed the role of SHP-1 in tumor progression, and a few studies have suggested that SHP1 plays a potential tumor suppressor role in various cancer types (106). Moreover, impaired function of SHP-1 has been shown to induce cancer progression by downregulating intracellular signaling transmembrane receptors, such as growth factor and cytokine receptors, leading to abnormal pathologies (107). Upregulation of SHP-1 activity induces cell apoptosis both *in vitro* and *in vivo* (54). In addition, Tai *et al* (54) reported that STAT3-related kinases or downstream effectors, including p-JAK1, p-JAK2, Mcl-1, and cyclin D1, are also induced in sorafenib-resistant cells. Evidence has shown that the JAK/STAT3 signaling pathway is a crucial modulator of the efficacy of sorafenib. Notably, decreased expression of SHP-1 was also observed in sorafenib-resistant cells. Collectively, the role of SHP-1-related STAT3 signaling in HCC has been verified; therefore, the SHP-1/STAT3 pathway may be an effective target for HCC treatment (54). SHP-1-mediated dephosphorylation of PKM2 at Y105 results in increased activity, and tetrameric PKM2 has reduced nuclear localization, which leads to the downregulation of the expression of oncogenic molecules, such as c-Myc and cyclin D1. Furthermore, constitutively active SHP-1 (D61A) can increase the percentage of tetrameric PKM2 and phosphorylation of PKM2 (Y105F), suggesting that SHP-1 determines the levels of dimeric/tetramer PKM2 and the subsequent nuclear localization via PKM2 Y105 dephosphorylation (108). SHP-1 (*PTPN6*), first identified in hematopoietic cells, is implicated in various hematopoietic signaling processes, including integration of immunoreceptor tyrosine-based activation motif-mediated inhibitory signals (109) and B-cell and natural killer (NK)-cell development (110,111). Furthermore, gankyrin is upregulated in chronic inflammation and induces STAT3 activation and IL-6 secretion by interacting with SHP-1 in non-parenchymal cells. Such proinflammatory interactions may induce the levels of stem cell markers in the tumor microenvironment and eventually promote HCC progression. Thus, the expression of gankyrin is defined as a promising predictor of the efficacy of advanced treatment for patients with HCC (56). Previously, gankyrin was demonstrated to enhance hepatocarcinogenesis via STAT3 activation through SHP-1 inhibition and IL-6 upregulation in the tumor microenvironment. Thus, STAT3/IL-6 signaling may involve gankyrin-regulated crosstalk between tumor cells and nonparenchymal cells (56).

PTPRO is a receptor type of PTP that has been defined as an integral membrane protein in numerous parenchymal cells (including lung, liver, and breast cells) (112,113). It has been previously demonstrated by the authors that PTPRO can suppress STAT3 activation, leading to reduced development of HCC. PTPRO can negatively regulate important pathways related to autophagy, such as the PI3K signaling pathway (63,114). Moreover, Zhang *et al* (115) reported that ptpro^−/−^ hepatocytes lead to the development of steatosis and induce tumorigenesis in mice fed a high-fat diet (HFD). PTPRO deletion significantly augmented obesity-reduced autophagy, as evidenced by increased p62 expression and a reduced LC3II/I ratio, as revealed by western blotting. Collectively, evidence confirmed that PTPRO deletion promotes obesity-related hyperinsulinemia and autophagy deficiency in the liver (Fig. 2C, Table III) (115). Furthermore, evidence suggested that PTPRO increases the cytoplasmic accumulation of p53 through the PI3K/Akt/MDM axis (115). Additionally, the expression of PTPRO has been demonstrated to be significantly reduced in HCC compared with normal tissues (63). PTPRO expression is suppressed *in vivo* in mice fed an HFD compared with that in mice fed a normal diet (116,117). PTPRO regulates autophagy and lipid metabolism in obesity and steatohepatitis (115). Furthermore, PTPRO regulates lipid metabolism through reduced expression of lipogenesis genes and induction of β-oxidation-related gene expression, and obesity significantly induces tumorigenesis in the liver in ptpro^−/−^ mice (115).

In the present review, extensive information was provided discussing whether PTPs play a critical role in inflammatory reactions and drug resistance to influence cancer progression in HCC. Numerous inflammatory cytokines/chemokines modulated by PTPs and several chemotherapeutic and targeted therapeutic drugs were illustrated, that are likely related to PTPs that play critical roles in numerous cellular mechanisms and signaling pathways. A total of 3 PTPs involved in HCC drug resistance were listed, including PTPN6, PTPN11 and PTPRO. In addition, there are six PTPs, PTPN6, PTPRD, CDC25A, PTPRO, PTPN11 and PTP1B. Among these, it was found that the three PTPs PTPN6, PTPN11 and PTPRO both induce drug resistance and alter inflammatory cytokine regulation, and these molecules can influence tumor growth and HCC progression. Hence, it was suggested that the three PTPs PTPN6, PTPN11 and PTPRO play equally important roles in HCC progression. The present review provided practical information for researchers to understand in an improved way the roles and functions of PTPs in cancer progression and hence may aid the identification of further therapeutic options to cure cancer.

According to findings from other groups about the roles of PTPs, it was suggested by the authors that PTPs play roles in inflammatory cytokines and drug resistance. In the future, it is considered by the authors that the effects of PTPs may be applied to clinical practice to evaluate whether PTP molecules could be useful predictors in patients with HCC. It was hypothesized that the levels of PTPs and inflammatory cytokines, such as IL-1β and IL-6, in HCC patients with chemotherapy/targeted drug resistance could be detected in tissues and plasma by immunohistochemistry and ELISA, respectively. Finally, the correlations between the levels of PTPs and IL-1β and IL-6 can also be analyzed to determine whether the correlations and these molecules could be markers to predict the prognosis and survival rate of drug-resistant patients.

## Figures and Tables

**Table I.** The subclasses of DSPs.

Subclass	Symbol	Member
Mitogen-activated protein kinase phosphatases	MPKs	11
Phosphatases of regenerating liver	PRLs	3
Phosphate and tension homologs	PTEN	5
Atypical DSPs		19
Myotubularins		16
CDC14s		4

DSPs, dual-specificity phosphatases.

**Table II.** Association between PTPs and cytokines.

Name	Mechanism	Pathway	Effect
PTPN6	Interaction with gankyrin ↑	STAT3 phosphorylation ↑	IL-6 secretion ↑
PTPRD	Correlation with PD-1 ↓	STAT3 pathway ↑	PD-L1 ↓
CDC25A	IL-6 and IL-1β ↓		Cell cycle arrest
PTPRO		JAK2/STAT3 ↓	IL-6 secretion ↑ PD-L1 ↓
PTPN11		RAS/ERK pathway/Integrin signaling ↑	HCC progression
PTP1B	IL-1β, IL-6 and TNF-α ↑	NF-κB pathway ↑	Inflammatory response ↑

↑, induction; ↓, reduction; PTP, protein tyrosine phosphatase; HCC, hepatocellular carcinoma; PTPRO, protein tyrosine phosphatase receptor type O.

**Table III.** The relationship between PTPs and drug resistance.

PTPs	Markers	Pathway	Effect
PTPN6	p-JAK1, p-JAK2, Mcl-1, and cyclin D1	JAK/STAT3 pathway ↑	Apoptosis ↑ HCC progression ↑
PTPN11	GADD45G	STAT3 pathway ↑	Senescence ↑
		MEK/ERK pathways ↑	Tumor growth ↓
		NF-κB pathway ↑	
		PI3K/AKT/mTOR pathway ↑	
PTPRO		STAT3 pathway ↓	HCC development ↓
	LC3II/I, p62	PI3K pathway ↓	Autophagy ↓

↑, induction; ↓, reduction; PTP, protein tyrosine phosphatase; p-, phosphorylated; HCC, hepatocellular carcinoma.

## Data Availability

Not applicable.
